# Electrospinning of Polyepychlorhydrin and Polyacrylonitrile Anionic Exchange Membranes for Reverse Electrodialysis

**DOI:** 10.3390/membranes11090717

**Published:** 2021-09-18

**Authors:** José A. Reyes-Aguilera, Liliana Villafaña-López, Elva C. Rentería-Martínez, Sean M. Anderson, Jesús S. Jaime-Ferrer

**Affiliations:** 1 División de Ciencias e Ingenierías, Universidad de Guanajuato, Loma del Bosque 103, León 37150, Mexico; jareyes@fisica.ugto.mx (J.A.R.-A.); renteriame2014@licifug.ugto.mx (E.C.R.-M.); 2 CIATEC A.C., Centro de Innovación Aplicada en Tecnologías Competitivas, Omega 201, León 37545, Mexico; lvillafana@ciatec.mx; 3 Information Systems, Wake Forest University, Winston-Salem, NC 27109, USA; anderss@wfu.edu

**Keywords:** electrospinning, anion exchange membrane, reverse electrodialysis, polyepichlorohydrin, polyacrylonitrile

## Abstract

The saline gradient present in river mouths can be exploited using ion-exchange membranes in reverse electrodialysis (RED) for energy generation. However, significant improvements in the fabrication processes of these IEMs are necessary to increase the overall performance of the RED technology. This work proposes an innovative technique for synthesizing anion exchange membranes (AEMs) via electrospinning. The AEM synthesis was carried out by applying a high voltage while ejecting a mixture of polyepichlorohydrin (PECH), 1,4-diazabicyclo [2.2.2] octane (DABCO^®^ 33-LV) and polyacrylonitrile (PAN) at room temperature. Different ejection parameters were used, and the effects of various thermal treatments were tested on the resulting membranes. The AEMs presented crosslinking between the polymers and significant fiber homogeneity with diameters between 1400 and 1510 nm, with and without thermal treatment. Good chemical resistance was measured, and all synthesized membranes were of hydrophobic character. The thickness, roughness, swelling degree, specific fixed-charge density and ion-exchange capacity were improved over equivalent membranes produced by casting, and also when compared with commercial membranes. Finally, the results of the study of the electrospinning parameters indicate that a better performance in electrochemical properties was produced from fibers generated at ambient humidity conditions, with low flow velocity and voltage, and high collector rotation velocity.

## 1. Introduction

Recent years have witnessed a boost in the search for renewable energy sources that are both eco-friendly and cost-effective. In this context, reverse electrodialysis (RED) technology has emerged as a promising candidate for generating electricity from the natural saline gradient present in river mouths [1]. The transport and stability properties of the ion-exchange membranes (IEMs) will determine the efficiency of the RED systems. To increase this performance, the IEM fabrication process should be improved to ensure membranes with higher mechanical stability and ion-transfer efficiency [2].

For over 30 years, the solution casting technique has been the traditional method for synthesizing anion exchange membranes (AEMs), which are basically conformed of a polymeric structure with fixed polar groups along the polymer chain. Polar groups are inserted into the main polymer chain by copolymerization of polar and nonpolar monomers [3]. The copolymerization reaction typically involves the chloromethylation of an aromatic polymer (typically a polysulfone) and its quaternization with a tertiary amine [3,4]. The polymerization reaction is carried out in a solvent, then poured over a surface for the final solvent evaporation [5].

An innovative technique for synthesizing IEMs by electrospinning has been recently proposed [6]; this method involves projecting a polymer solution through a nozzle under a high potential difference (normally in the kV range) between the nozzle and a surface positioned some distance away. The solution will form small droplets that are electrically repulsed and become elongated to form fibers; these fibers are collected on the surface and comprise the final membrane. This technique is promising for the synthesis of IEMs with desirable properties.

In practice, optimizing the polymer solution composition and adjusting the feed velocity, the applied voltage, the rotational speed of the collector and the distance between the collector and the ejector tip allow for a fine-tuning of the structure and properties of the membrane, including mean nanofiber diameter, swelling degree (SD), hydrophobicity, ion-exchange capacity (IEC) and more [3,6,7,8]. Some of these properties can be linked, such as the SD and the IEC [9], therefore a careful balance must be made between the mechanical stability and the overall IEC.

The literature features a wide variety of potential applications for electrospun membranes; namely, absorption of metals and ionic impurities [10], wastewater treatment [11], anti-bacterial treatments [12], neural tissue engineering [13], fuel cells and reverse electrodialysis [14,15]. The polymers and solvents used to synthesize the membranes are diverse and highly specific to the desired application; thus, selecting the operating parameters during membrane fabrication is a highly complex problem [10,11,12,14,16]. Nonetheless, once the precursor materials of the membrane have been selected, it is possible to determine the impact on the final membrane characteristics of each one of the operational parameters [17]. These studies tend to primarily focus on the morphology of the membranes employed for dialysis and fuel cells, and, thus, are limited to aspects such as the average diameter of the obtained fibers, porosity and the presence of defects in the fibers [14].

On the other hand, there are very few published works on RED that analyze the ion-exchange capacity and its relation to membrane swelling from water absorption; these factors are directly related and have a negative relationship in synthesized membranes from solutions of PECH, PAN and DABCO in solvents such as DMSO and DMF [18]. The effect of the applied voltage and of the tip-to-collector distance on the ion-exchange capacity and the swelling degree of a membrane has been studied previously [19]; these properties are highly interrelated and are of great importance to the overall membrane performance.

In this work, a method of IEM synthesis via electrospinning is proposed for RED applications, where the crosslinking between the PECH, PAN and DABCO^®^ 33-LV improves the integrity and mechanical stability of the membrane. A rotating collector surface was used to align the fibers; thermal treatments were applied and the influence of the different operating parameters on the morphology of the membrane was verified. Additionally, an exhaustive characterization of AEM properties such as the fixed-charge density, the ion-exchange capacity and the swelling degree was carried out, as these are crucial factors that impact the overall permselectivity of the membrane.

## 2. Materials and Methods

### 2.1. Reagents

All reagents used were acquired from Sigma Aldrich, Saint Louis, MO, USA. PECH (Mw ≈ 700,000 g/mol) was used as the active polymer and PAN (Mw ≈ 150,000 g/mol) was used as the inert polymer. DABCO^®^ 33-LV with a Mw ≈ 112.17 g/mol and a density (ρ) of 1.02 g/mL was used as a reticulating agent. Dimethylformamide (DMF ≥ 99.8%) with ρ = 0.98 g/mL and a boiling point of 153 °C, and dimethyl sulfoxide (DMSO ≥ 99.5%, GC) with ρ = 1.10 g/mL and a boiling point of 189 °C were used as solvents.

### 2.2. Membrane Preparation

A first study of the AEMs was carried out using membranes synthesized from solutions of PECH, PAN and DABCO in two different solvents, DMSO and DMF; Table 1 presents the quantities and ratios that were used to prepare these. The solutions were separately agitated at room temperature and constant speed (400 rpm) for 12 h. After this time, the three solutions were combined in the proportions shown in Table 1 and were heated under reflux at 80 °C for 30 min. The final solution was left to cool at room temperature and under constant agitation.

The AEMs were produced using the semi-professional Ne100 Nanospinner electrospinning machine, INOVENSO Ltd. Istanbul, Turkey. The tip-to-collector distance was set to 17 cm, and the humidity of the chamber was maintained between 65–75%. A drum cylinder rotated at 100 rpm and covered with a 20 µm aluminum sheet was used as fiber collector. The aluminum sheet was submerged in deionized water in order to facilitate the removal of the membrane. The three AEMs were cut into two equal parts; one part was characterized with no further treatment (NT) and the other was heat treated before being measured (HT). The heat treatment consisted of heating the membrane on a glass plate in a convection oven for 2 h at 110 °C followed by 130 °C for 30 min. Every AEM underwent a visual supervision after being submerged in aqueous solutions at different pH values (from 1 to 14) for a duration of 12 h. This was done in order to verify the structural stability of the synthesized AEMs.

The second study used DMF as the solvent, and various parameters of the electrospinning process were varied; namely, the flow velocity of the injected polymer solution and the collector rotation speed. The effects of the ambient humidity, the pre-treatment of the polymer solutions and the thickness of the resulting membranes were also analyzed. Table 2 shows the experimental parameters that remained fixed during this study. Similar to the first study, the AEM polymer solution received a distillation pre-treatment with a reflux condenser at 80 °C for 90 min before the synthesis. The generated AEMs were characterized by their thickness, SD, IEC, permselectivity, fixed-charge density and electrical resistance.

The prepared membranes were compared with the AMX and Type X AEM commercial membranes. The AMX is a homogeneous membrane from Neosepta, ASTOM Corporation, Tokio, Japan. This membrane uses a copolymer of polystyrene and divinylbenzene as its ion exchange matrix. The Type X AEM is a homogeneous membrane manufactured by Fujifilm, Tilburg, The Netherlands that uses polyamide as its ion exchange matrix. Some of the characteristics of these membranes are presented in Table 3.

### 2.3. Membrane Characterization

The details of the different techniques used for the membrane characterization were reported previously [19,21].

#### 2.3.1. Scanning Electron Microscopy (SEM)

The prepared AEMs were observed using two different field emission scanning electron microscopes (FE-SEMa Carl Zeiss SIGMA-HDVP, Oberkochen, Germany and a JEOL JSM-7800F system, Osaka, Japan). Both units were equipped with an Energy Dispersive System (EDS).

#### 2.3.2. Fourier Transform Infrared Spectroscopy (FTIR)

FTIR spectra were obtained using a Thermo ScientificTM Nicolet TM iS10 FTIR spectrometer, Waltham, MA, USA. The chemical homogeneity of the prepared AEMs was confirmed on both sides of the membrane.

#### 2.3.3. Atomic Force Microscopy (AFM)

The roughness of the synthesized membranes was determined by measuring the root mean square (RMS) surface. The membrane analysis was carried out in intermittent contact mode, and the measured area was 98.182 × 92.945 µm on all samples. The dry membranes were dried at 35 °C for 48 h, and the semi-humid membranes were submerged in 25 mL of deionized water for 7 min, with the excess water removed prior to analysis.

#### 2.3.4. Swelling Degree (SD)

The SD is an indicator of the amount of water that is incorporated into the bulk of the membrane when it is exposed to it. In practice, the increase in weight of the wet membrane is determined with respect to the dry membrane, and is expressed as follows [19]:

(1) $$\mathrm{SD}\;\left(\%\right)=\frac{w_{\mathrm{wet}}-{\;w}_{\mathrm{dry}}}{w_{\mathrm{dry}}}\times 100$$

where w_wet_ = hydrated membrane weight (mg) and w_dry_ = dried membrane weight (mg).

#### 2.3.5. Ion-Exchange Capacity (IEC)

The IEC expresses the relationship between the number of charges per mass on the dry membrane. This value is obtained via titration, and is used in the following:

(2) $$\mathrm{IEC}\;\left(\frac{\mathrm{meq}}{g}\right)=\frac{V_{t}\times C_{t}}{w_{\mathrm{dry}}}$$

where V_t_ = titrant volume consumed (L) and C_t_ = titrant concentration (mol/L). The specific pre-treatment of the IEMs and the different solution used for the titration technique are described in detail in reference [21].

#### 2.3.6. Fixed Charge Density (CD_fix_)

The CD_fix_ is defined as the ratio between milliequivalents of fixed groups per volume of water in the membrane, and can be calculated as [22]:

(3) $${\mathrm{CD}}_{\mathrm{fix}}=\frac{\mathrm{IEC}}{\mathrm{SD}}$$

where the IEC and SD are the parameters defined above.

#### 2.3.7. Water Contact Angle (θ_w_)

The θ_w_ in sessile drop mode was measured with a DataPhysics OCA 15EC, Filderstadt, Germany. The methods used here for the specific pre-treatment and preparation of the IEMs are described in detail in references [19,21].

#### 2.3.8. Thickness

In order to measure the thickness of the synthesized AEMs, the membranes were annealed for 48 h at 35 °C and then placed in a desiccator for an additional 24 h. The dried membranes were glued to a glass slide using double-sided tape, and their cross-section was photographed using a DataPhysics OCA 15EC instrument. The thickness was determined by analyzing the photographs with the FIJI software package, Bethesda, Maryland, USA [23]. Ten photographs were taken from different sections of the membranes in order to verify the homogeneity, with ten separate measurements to minimize the uncertainty.

#### 2.3.9. Permselectivity

The permselectivity (S) describes the ability of the membranes to limit the co-ion transport within the membranes, and it is a key parameter in RED applications. Different methodologies have been reported to determine S, the most used being the measurement of the potential difference of two solutions at different concentrations separated by the membrane of interest. Equation (4) was used to obtain S values:

(4) $$S\;\left(\%\right)=\frac{\Delta E_{\exp}}{\Delta E_{\mathrm{th}}}$$

where ∆E_exp_ is obtained with the same experimental protocol detailed in [21] and ∆E_th_ was calculated from the Nernst equation.

#### 2.3.10. Electrical Resistance

Membrane electrical resistance (ER) was obtained with the same methodology described in [24] and following the Equation (5):

(5) $$\mathrm{ER}\;\left({\Omega \;\mathrm{cm}}^{2}\right)=\left(R_{m+s}-R_{m}\right)\times A$$

where *R_m+s_* is the solution and membrane resistance (Ω), *R_m_* is the membrane resistance (Ω) and *A* is the membrane surface (cm^2^). To determine the resistance of the membrane, the impedance was measured in an H cell with and without a membrane. Since the membrane is in contact with a liquid electrolyte, a four-electrode setup helps to eliminate the contribution of the electrode injecting stimulus/electrolyte charge transfer resistance from the impedance spectra, focusing the probing on the membrane and its interfaces. The real-axis intercept at high frequencies yields the value of $R_{m}$ or $R_{m+s}$. The electrolyte resistance is obtained by first following the procedure without the membrane, and then subtracting its contribution to isolate the effective membrane resistance [24].

## 3. Results and Discussion

Chemical resistance tests at various pH values and different times (24, 72 and 168 h) were performed on the synthesized AEMs. High stability was a characteristic of all the AEMs. No physical damage was observed in the pH range of 2 to 12; however, above and below these pH values, the AEMs presented color changes and less physical integrity.

### 3.1. Morphology

**Figure 1:**
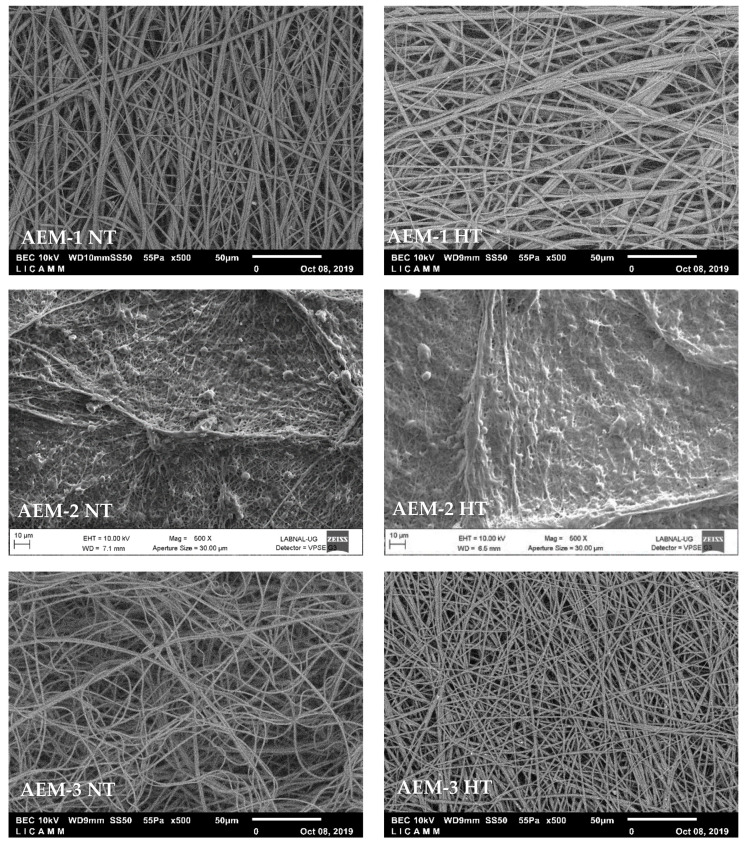
Scanning electron microscopy (SEM) images of the surface of the anion exchange membranes (AEMs) without heat treatment (NT, left side) and with heat treatment (HT, right side) with a magnification of ×500.

SEM images were analyzed in order to elucidate the structure of the electrospun fibers in the AEMs. Figure 1 presents the images of the membrane surfaces for AEMs without heat treatment (NT, left side) and with heat treatment (HT, right side) with a magnification of ×500. AEM-1 and AEM-3 presented disordered fibers, while AEM-2 had a dense layer of melted fibers; this layer was possibly caused by an incomplete evaporation of the solvent, as AEM-2 had 16 times more solvent than the active polymer, and 20 times more solvent than the inert polymer. Since AEM-2 lacked distinct fiber formation, the rest of the study was only carried out on AEM-1 and AEM-3.

**Figure 2:**
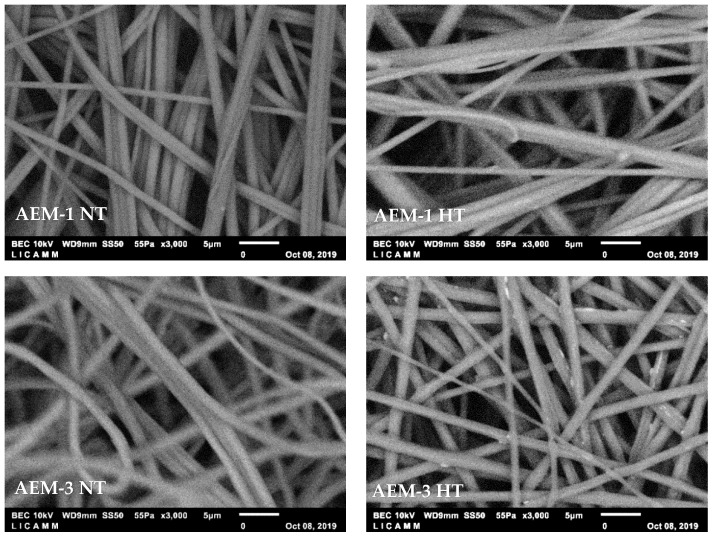
SEM images of the surface of the AEMs without heat treatment (NT, **left** side) and with heat treatment (HT, **right** side) with a magnification of ×3000.

SEM images of each AEM-1 and AEM-3 were also taken at a magnification of ×3000. This allowed for closer inspection of the morphology of the membranes and for determining the fiber diameter. Figure 2 presents these images for NT (left side) and HT (right side) membranes. AEM-1 presents fiber bundles, a defect that is formed during the fiber formation stage [24]. AEM-3 has both fiber bundles and loose fibers; these being significantly reduced in the HT membrane. Due to the many defects in AEM-1, the remaining sections of this work will only focus on AEM-3; the NT membrane will thus be referred to as AEM-NT, and the HT membrane as AEM-HT.

The fiber diameter was determined from these images, and these values are reported in Table 4 along with the volume of polymer solution, thicknesses, and areas for both the NT and HT membranes. Notably, an AEM with 476 cm^2^ of area was synthesized using only 3 mL of polymer solution. The fiber diameter was not significantly impacted by the heat treatment and remained well above the 100 nm limit that is required to be considered true nanofiber [25].

### 3.2. Membrane Structure and Roughness

**Figure 3:**
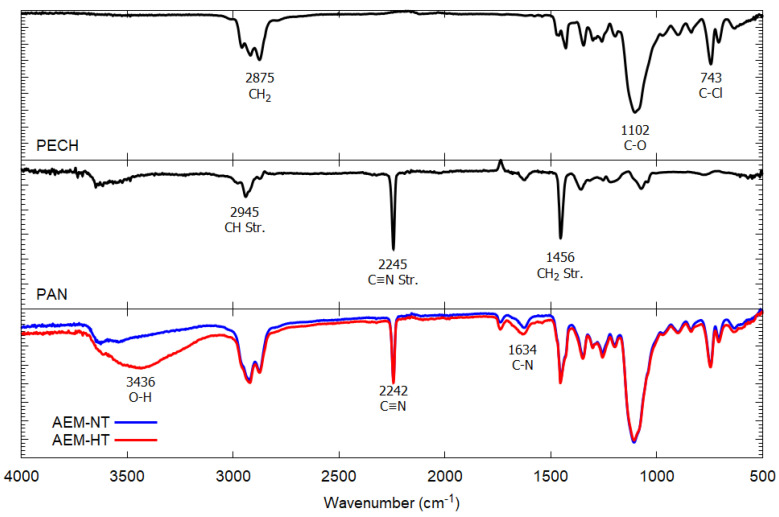
PECH, PAN, AEM-NT and AEM-HT FTIR spectrums.

The upper and middle panels of Figure 3 show the characteristic peaks of the PECH and PAN samples. The lower panel presents the spectrum of the synthetized membrane where three peaks are observed at 3436 cm^−1^, 2242 cm^−1^ and 1634 cm^−1^, which correspond with the OH group of PECH, the C-N group of PAN and the C-N group of PECH, respectively [18]. The presence of the three peaks in the spectrum of the synthesized membrane is evidence of a good cross-linking between the polymers. On the other hand, the heat treatment does not have a significant impact on the structure of the obtained membrane, as seen in the AEM-NT and AEM-HT FTIR spectra.

**Figure 4:**
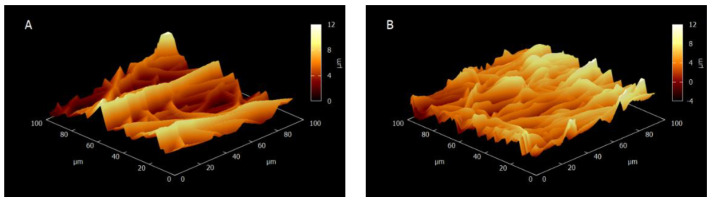

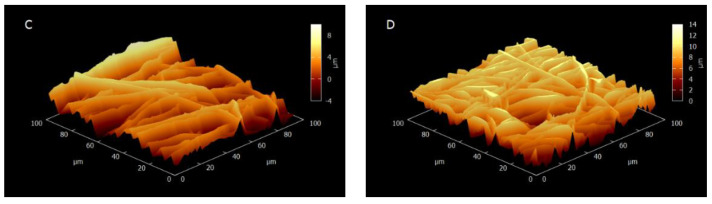
Atomic force microscopy (AFM) images for the electrospun AEMs. (**A**) dry AEM-NT, (**B**) dry AEM-HT, (**C**) semi-humid AEM-NT and (**D**) semi-humid AEM-HT.

The membrane was also studied using AFM. Both the NT and HT samples were measured in dry and semi-humid states, as shown in Figure 4; likewise, Table 5 presents the roughness values calculated from these images. The NT membranes were less swollen than the HT membranes, which was corroborated by the root mean square roughness values shown in Table 5, where the HT value is higher than the NT value. The fiber fraction (the inverse of the air volume between the fibers) and the roughness are proportional [26]; this suggests that there were more fibers in the AEM-HT than in the AEM-NT as confirmed by the numerous hills and valleys shown in Figure 4. Likewise, the additional roughness also corresponds with an increase in the hydrophobicity, since more air bubbles can get caught within the fiber mesh, causing an increase in the water contact angle [27].

### 3.3. Swelling Degree (SD), Ion-Exchange Capacity (IEC), Fixed Charge Density (CD_fix_), Contact Angle, and Membrane Thickness

Table 6 presents data for the SD, the IEC, the CD_fix_, the contact angles and the membrane thickness of the synthesized AEMs compared to membranes prepared by casting with the same polymer solution [21] and also AMX and Type X AEM commercial membranes [20]. The SD values for membranes obtained by electrospinning are lower than those reported for membranes obtained by the casting solvent technique [21] and for commercial membranes [20]; conversely, the IEC and CD_fix_ were much larger. These findings suggests that the electrospun membranes have an increased performance in terms of permselectivity, due to an increase in available sites in the polymer matrix for charge transfer [19].

A contact angle greater than 90° is indicative that the electrospun and casted membranes were hydrophobic, as expected, since the AEMs have positive charges on their surfaces. The contact angle is higher for the electrospun membranes due to the air bubbles that were caught within the fiber mesh. The thickness of the AEM-NT is similar to the commercial membranes, while the AEM-HT thickness is closer to the casted membranes. The membrane thickness decreased after heat treatment; this was probably caused by the membrane becoming tensioned after adhering to the glass plate within the convection oven. The effects of the heat and the tension cause the molecular chains of the fibers to stretch and rearrange themselves in the direction of the tension, thus reducing the overall thickness [28]. This reduction in thickness for the AEM-HT makes it more favorable for ion-transport [21].

### 3.4. Influence of the Thickness over the AEM-HT Properties

**Figure 5:**
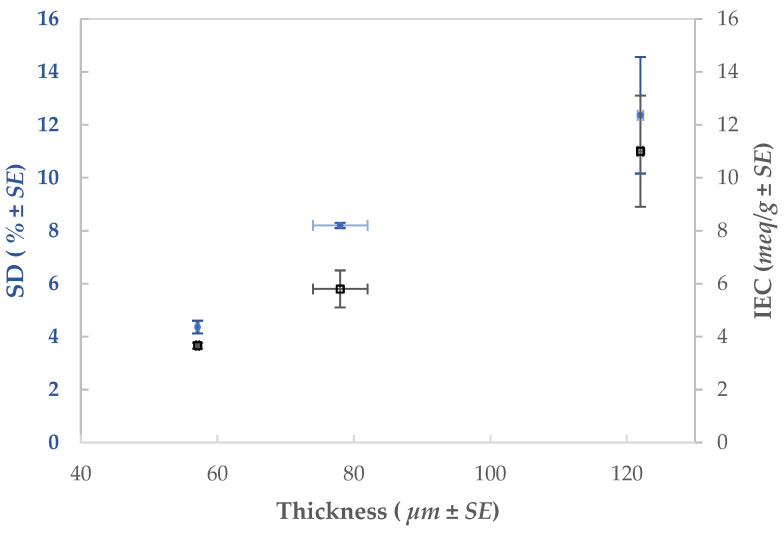
SD and IEC as functions of the membrane thickness for the AEM-HT baseline membrane.

The membrane thickness has a significant impact on the SD and IEC of the synthesized membranes [21]. In Figure 5, it can be seen that both parameters increase proportionally with the thickness.

The increase in the IEC is due to the larger available surface area from the nanometric effect of the electrospun membrane, which allows for an increase in the quantity of ion-exchange groups [14]. In turn, the ionic transfer is faster for thinner membranes which improves the permselectivity and yields a smaller ER (Table 7).

Section 3.3 showed that the AEM-HT membrane had a reduced swelling degree by 75% over the membrane synthesized by casting (Table 6). Additionally, it is possible to modulate the SD of the membrane with the thickness; the thicker the membrane, the larger the surface area and the more water that can be incorporated into the body of the membrane.

### 3.5. Influence of the Reflux Distillation on the AEM-HT Properties

Table 8 shows the membrane properties without the reflux distillation of the polymeric solution. The results show that the pre-treatment of the polymeric mixture ensures the crosslinking between the PECH, PAN and DABCO, which improves the membrane properties.

### 3.6. Trends in the AEM Properties as Functions of Humidity, Flow Velocity, and Collector Speed

Since both the AEM-NT and AEM-HT had better properties than the casted and commercial membranes, a final study was carried out to produce membranes with enhanced performance by tuning the electrospinning parameters. Special care was taken to produce membranes thinner than 60 µm, since this thickness range was shown to have better S and ER values. Table 9 presents the studied parameters with the different experimental conditions and their effect on the AEM properties. All experiments were done in triplicate; the average values with standard error (SE) are reported and the best result has been highlighted at 300 rpm of collector speed.

No significant improvement in the membrane properties was detected when increasing the humidity; however, a slight increase in the SD and IEC were observed. This could possibly be attributed to the surface of the fibers becoming more irregular and textured at the moment of phase separation; in effect, the increase in atmospheric humidity causes a reduction in the phase separation time due to the accumulation of water in the jet of polymer solution. This leads to the formation of cavities on the surface of the fibers and an increase in surface area [29].

The flow velocity of the polymeric mixture enhances the S and ER properties of the membrane at around 0.225 mL/h, although the IEC has a slight decrease at higher velocities. Effectively, high flow velocities generate defects in the membrane that impact its electrochemical performance. Contrarily, low velocities reduce the diameter of the produced fibers which increases the surface area; this has been shown to enhance the IEC [14].

**Figure 6:**
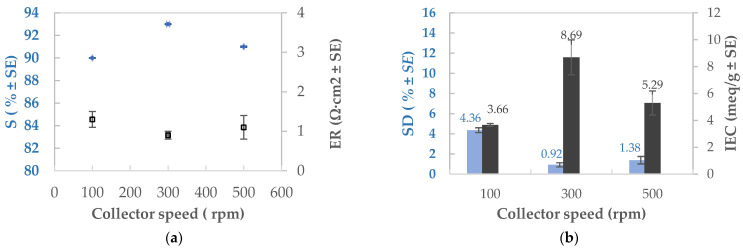
Influence of the collector velocity on the AEM properties: (**a**) SD and IEC; (**b**) S and ER.

Lastly, the collector velocity has significant influence over the membrane properties, as shown in Figure 6a,b. An optimal value of 300 rpm was observed for obtaining a low SD and a high IEC. Increasing the collector velocity enhances the alignment of the fibers which positively impacts the SD and IEC. The improved fiber arrangement increases the surface area; coupled with the hydrophobic character of the membrane, the water-receiving channels are also reduced. The fiber alignment generates a better distribution of the ion groups, improving the selectivity of the membrane (higher S) that is a function of the relationship between the SD and the IEC (CD_fix_). Membrane resistance to the ionic flow is also reduced.

## 4. Conclusions

Three AEMs were synthesized using different solvents, polymer rations and thermal treatments. The membrane produced with DMF had the best structural properties, with good cross-linking between the polymers, good chemical resistance and an adequate contact angle. The electrospun membrane presented superior SD, IEC and CD_fix_ values when compared with casted and commercial membranes, suggesting an enhanced permselectivity performance. The heat treatment yielded a thinner membrane, which could be favorable for ion-transfer.

It was demonstrated that the physicochemical properties of the AEMs could be controlled by fine-tuning the different electrospinning parameters. These properties were enhanced when working in low-humidity conditions and with a high collector rotation speed, coupled with low flow velocity and voltage to avoid spurious fiber defects. Likewise, membranes thinner than 100 µm also had improved characteristics. These results suggest that electrospinning is a very promising technique for synthesizing IEMs, allowing for a large degree of control over the final membrane properties. These parameters allowed us to obtain electrospun membranes with a permselectivity value of 93% and an electrical resistance of 0.9 Ω·cm^2^.

## Figures and Tables

**Table 1:** Anion exchange membrane (AEM) preparation parameters.

AEM	Solvent	PECH:Solvent (*w:w*)	PAN:Solvent (*w:w*)	DABCO:Solvent (*w:w*)	PECH:PAN:DABCO (*w:w:w*)	Flow (mL/h)	Voltage (kV)
1	DMSO	20:80	12:88	12.5:88	2:1:2	0.1	30
2	DMF	2.5:40	1.5:30	1.53:11	2:1:2	0.1	14
3	DMF	20:80	12:88	12.5:88	1:2:0.83	0.225	15

**Table 2:** Fixed electrospinning parameters that were used in this study as a baseline.

Parameter	Value
PECH:PAN:DABCO	1:2:0.83 (*w:w:w*)
Humidity	60%
Temperature	22 ± 1 °C
Applied voltage	15 kV
Polymer solution volume	6 mL
Flow velocity	0.225 mL/h
Collector speed	100 rpm
Tip-to-collector distance	17 cm

**Table 3:** Characteristics of the commercial membranes used as reference in this study.

Commercial Membranes	Swelling Degree (%)	Ion-Exchange Capacity (meq/g)	Fixed Charge Density (meq/g)	Thickness (µm)
AMX [20]	14 ± 2 16	1.23 ± 0.05 1.25	7.8–9.8	125 ± 5
Type X AEM [20]	23 ± 2	1.50 ± 0.05	6.2–6.9	115 ± 5 125

**Table 4:** Volume of polymer solution used for the synthesis of AEM-NT and AEM-HT, along with the obtained area, fiber diameter and membrane thickness.

Volume (mL)	Area (cm^2^)	Treatment	Diameter ± SE (nm)	Thickness ± SE (µm)
3	476	NT	1510 ± 70	130 ± 4
		HT	1400 ± 50	78 ± 4

SE: Standard error of 3 independent measurements.

**Table 5:** Root mean square (RMS) roughness values for the electrospun AEM-NT and AEM-HT membranes.

Membrane	Procedure	RMS Roughness (µm)
AEM-NT	Dry	1.83
	Semi-humid	1.91
AEM-HT	Dry	2.07
	Semi-humid	2.15

**Table 6:** Values for swelling degree (SD), ion exchange capacity (IEC), fixed charge density (CD_fix_), contact angles (θ_w_) and membrane thickness of the synthesized AEMs, compared to casted and commercial membranes.

Membrane	SD ± SE (%)	IEC ± SE (meq/g)	CD_fix_ ± SE (meq/g)	θ_w_ ± SE (°)	Thickness (µm)
AEM-NT	8.5 ± 0.6	5.3 ± 0.3	62.2 ± 4.2	121 ± 4	130 ± 4
AEM-HT	8.2 ± 0.1	5.8 ± 0.7	70.0 ± 8.3	127 ± 2	78 ± 4
AEM-HT casting	30.1 ± 1.1	1.4 ± 0.1	4.5 ± 0.4	111 ± 2	77 ± 3
AMX [20]	14 ± 2 16	1.23 ± 0.05 1.25	7.8–9.8	--	125 ± 5
Type X AEM [20]	23 ± 2	1.50 ± 0.05	6.2–6.9	--	115 ± 5 125

**Table 7:** Values for SD, IEC, CD_fix_, permselectivity (S) and membrane electrical resistance (ER) in function of membrane thickness.

Parameter	Value	Thickness ± SE	SD ± SE	IEC ± SE	CD_fix_ ± SE	S ± SE	ER ± SE
		(µm)	(%)	(meq/g)	(meq/g)	(%)	(Ω·cm^2^)
Thickness	<100 µm	57.1 ± 0.2	4.36 ± 0.24	3.66 ± 0.11	83.94 ± 0.29	90 ± 0.02	1.3 ± 0.2
		78 ± 4	8.2 ± 0.1	5.8 ± 0.7	70.0 ± 8.3	--	--
	>100 µm	122 ± 0.4	12.36 ± 2.2	11.00 ± 2.1	88.99 ± 0.03	88 ± 0.1	2.5 ± 0.5

**Table 8:** Influence of the polymeric mixture pre-treatment on the membrane properties.

Parameter	Thickness ± SE	SD ± SE	IEC ± SE	CD_fix_ ± SE	S ± SE	ER ± SE
	(µm)	(%)	(meq/g)	(meq/g)	(%)	(Ω·cm^2^)
Without refluxdistillation	61.4 ± 0.3	3.43 ± 0.07	2.70 ± 0.03	78.71 ± 0.13	87 ± 0.03	3.5 ± 0.02
With reflux distillation	57.1 ± 0.2	4.36 ± 0.24	3.66 ± 0.11	83.94 ± 0.29	90 ± 0.02	1.3 ± 0.2

**Table 9:** Influence of the humidity, flow velocity and collector speed on the AEM properties.

Parameter	Value	Thickness ± SE	SD ± SE	IEC ± SE	CD_fix_ ± SE	S ± SE	ER ± SE
		(µm)	(%)	(meq/g)	(meq/g)	(%)	(Ω·cm^2^)
Humidity	60%	57.1 ± 0.2	4.36 ± 0.24	3.66 ± 0.11	83.94 ± 0.29	90 ± 0.02	1.3 ± 0.2
	80%	54.3 ± 0.2	4.41 ± 0.03	4.39 ± 0.4	99.54 ± 0.35	89 ± 0.01	1.4 ± 0.04
Flow velocity	0.225 mL/h	57.1 ± 0.2	4.36 ± 0.24	3.66 ± 0.11	83.94 ± 0.29	90 ± 0.02	1.3 ± 0.2
	0.325 mL/h	42.9 ± 0.1	4.53 ± 0.11	3.95 ± 0.02	87.19 ± 0.09	87 ± 1.3	1.9 ± 0.3
	0.525 mL/h	64.3 ± 0.4	10.77 ± 0.04	4.33 ± 0.2	40.22 ± 0.16	85 ± 0.4	2.2 ± 0.02
Collector speed	100 rpm	57.1 ± 0.2	4.36 ± 0.24	3.66 ± 0.11	83.94 ± 0.29	90 ± 0.02	1.3 ± 0.2
	300 rpm	41.4 ± 0.5	0.92 ± 0.21	8.69 ± 1.3	944 ± 0.3	93 ± 0.04	0.9 ± 0.1
	500 rpm	51.4 ± 0.2	1.38 ± 0.37	5.29 ± 0.9	383 ± 0.64	91 ± 0.05	1.1 ± 0.3

## Data Availability

Data presented in this study is contained within the article.
